# Supplementary material for: Natural History of the Genus Elasmoderus Saussure, 1888 (Orthoptera: Tristiridae), an Endemic and Eremic Element of the Transitional Coastal Desert of Chile
Source: Insects. 2024 Jul 9;15(7):513. doi: 10.3390/insects15070513 (PMC11277456; doi:10.3390/insects15070513)
Supplement: Supplementary file 1 [file insects-15-00513-s001.zip › insects-2963390-supplementary.pdf]

Supplemental Table S1. Distributional records for species of the genus *Elasmoderus* (Orthoptera: Tristiridae).

ID	Species	Location	Province	Region	Latitude	Longitude	Habitat	References	Registration date
1	*Elasmoderus lutescens* (Blanchard, 1851)	Copiapó	Copiapó	Atacama	27.3530	70.3238	Coastal desert scrub	Cigliano et al. (1989), Elgueta et al. (1999)	December 1940
2	*Elasmoderus lutescens* (Blanchard, 1851)	Chanarcillo	Copiapó	Atacama	27.3716	70.3419	Coastal desert scrub	Cigliano et al. (1989), Elgueta et al. (1999)	December 1965
3	*Elasmoderus lutescens* (Blanchard, 1851)	Quebrada del Morel	Copiapó	Atacama	27.6293	70.4751	Flowery desert of the Llanos	Data published in this work	-
4	*Elasmoderus lutescens* (Blanchard, 1851)	Punta Chasco	Copiapó	Atacama	28.4252	70.6255	Flowering desert	Data published in this work	-
5	*Elasmoderus lutescens* (Blanchard, 1851)	Sarco	Copiapó	Atacama	28.4267	70.6355	Flowery desert of the Llanos	Data published in this work	-
6	*Elasmoderus lutescens* (Blanchard, 1851)	Caserones	Copiapó	Atacama	27.3557	70.6692	Flowery desert of the Llanos	Elgueta et al. (1999)	-
7	*Elasmoderus lutescens* (Blanchard, 1851)	Castilla	Copiapó	Atacama	27.9046	70.6839	Coastal desert scrub	Elgueta et al. (1999)	-
8	*Elasmoderus lutescens* (Blanchard, 1851)	Ramadilla	Copiapó	Atacama	28.1130	69.7497	Native vegetation, no attack on crops reported	Cepeda-Pizarro et al. (2006)	November 1970
9	*Elasmoderus lutescens* (Blanchard, 1851)	Quebrada Honda	Huasco	Atacama	27.9909	71.1271	Xerophytic scrub	Elgueta et al. (1999)	-
10	*Elasmoderus lutescens* (Blanchard, 1851)	Carrizal Bajo	Huasco	Atacama	28.0851	71.1508	Flowery desert of the Llanos	Cigliano et al. (1989), Elgueta et al. (1999)	-
11	*Elasmoderus lutescens* (Blanchard, 1851)	Parque Nacional Llanos de Challe	Huasco	Atacama	28.2018	71.1437	Flowery desert of the Llanos	Data published in this work	October 2015
12	*Elasmoderus lutescens* (Blanchard, 1851)	Tres Playitas	Huasco	Atacama	28.4025	71.1875	Flowery desert of the Llanos	Data published in this work	October 2015
13	*Elasmoderus lutescens* (Blanchard, 1851)	Huasco	Huasco	Atacama	28.4732	71.2184	Coastal desert scrub	Elgueta et al. (1999), Alfaro et al. (2013)	-
14	*Elasmoderus lutescens* (Blanchard, 1851)	Vallenar	Huasco	Atacama	28.5755	70.7327	Interior desert scrub	Cigliano et al. (1989), Elgueta et al. (1999)	September 1957
15	*Elasmoderus lutescens* (Blanchard, 1851)	S de Vallenar	Huasco	Atacama	28.6245	70.7516	Interior desert scrub	Cigliano et al. (1989)	November 1972
16	*Elasmoderus lutescens* (Blanchard, 1851)	Domeyko	Huasco	Atacama	28.9583	70.8880	Flowery desert of the Serranias	Cigliano et al. (1989), Elgueta et al. (1999)	-
17	*Elasmoderus lutescens* (Blanchard, 1851)	Llanos de Pajonales	Huasco	Atacama	28.1923	71.1507	Flowery desert of the Serranias	Data published in this work	October 2015
18	*Elasmoderus lutescens* (Blanchard, 1851)	Pajonales	Huasco	Atacama	28.9583	70.8880	Flowery desert of the Serranias	Data published in this work	October 2015
19	*Elasmoderus lutescens* (Blanchard, 1851)	El Algarrobo	Huasco	Atacama	28.6633	70.9048	Flowery desert of the Serranias	Elgueta et al. (1999)	-
20	*Elasmoderus lutescens* (Blanchard, 1851)	Desierto costero del Huasco	Huasco	Atacama	28.4732	71.2184	Coastal desert scrub	Alfaro et al. (2013)	-
21	*Elasmoderus lutescens* (Blanchard, 1851)	Fundo Marañon	Huasco	Atacama	28.5199	70.7411	Coastal desert scrub	Elgueta et al. (1999)	-
22	*Elasmoderus lutescens* (Blanchard, 1851)	Chañaral de Aceituno	Huasco	Atacama	29.0444	71.4197	Native vegetation	Cigliano et al. (1989), Elgueta et al. (1999)	-
23	*Elasmoderus lutescens* (Blanchard, 1851)	El Carrizo	Huasco	Atacama	29.0502	70.4962	Native vegetation and crops slightly affected	Cepeda-Pizarro et al. (2006)	1975
24	*Elasmoderus lutescens* (Blanchard, 1851)	Cuesta de Pajonales	Vallenar	Atacama	29.1488	70.9954	Coastal desert scrub	Cigliano et al. (1989), Elgueta et al. (1999)	-
25	*Elasmoderus lutescens* (Blanchard, 1851)	Incahuasi	Elqui	Atacama	29.2288	71.0120	Coastal desert scrub	Cigliano et al. (1989), Elgueta et al. (1999)	November 1957
26	*Elasmoderus lutescens* (Blanchard, 1851)	El Tofo	Elqui	Coquimbo	29.4560	71.2427	Coastal native vegetation	Cigliano et al. (1989), Elgueta et al. (1999)	November 1972 - Described as *Elasmoderus rabiosus*
27	*Elasmoderus lutescens* (Blanchard, 1851)	Cuesta de La Higuera	Elqui	Coquimbo	29.5078	71.2160	Coastal desert scrub	Elgueta et al. (1999)	-

28	*Elasmoderus lutescens* (Blanchard, 1851)	Cuesta de Los Hornos	Elqui	Coquimbo	29.6305	71.2813	Coastal desert scrub	Elgueta et al. (1999)	-
29	*Elasmoderus lutescens* (Blanchard, 1851)	Condoriaco	Elqui	Coquimbo	29.7061	70.8361	Coastal desert scrub	Elgueta et al. (1999)	-
30	*Elasmoderus lutescens* (Blanchard, 1851)	Llanos de Arqueros, Condoriaco	Elqui	Coquimbo	29.7063	70.8374	Coastal desert scrub	Cigliano et al. (1989)	September 1957
31	*Elasmoderus lutescens* (Blanchard, 1851)	Llanos del Potroso, Condoriaco	Elqui	Coquimbo	29.7063	70.8374	Coastal desert scrub	Cigliano et al. (1989)	October 1958
32	*Elasmoderus lutescens* (Blanchard, 1851)	Cuesta La Viñita, Condoriaco	Elqui	Coquimbo	29.8242	70.8213	Coastal desert scrub	Cigliano et al. (1989)	October 1955
33	*Elasmoderus lutescens* (Blanchard, 1851)	Quebrada Grande	Elqui	Coquimbo	29.4735	70.9739	Native vegetation, no attack on crops reported	Cepeda-Pizarro et al. (2006)	September 1974
34	*Elasmoderus lutescens* (Blanchard, 1851)	Norte cuesta de Los Hornos	Elqui	Coquimbo	29.5112	71.2117	Native vegetation	Cigliano et al. (1989)	-
35	*Elasmoderus lutescens* (Blanchard, 1851)	Cuesta de Los Hornos	Elqui	Coquimbo	29.6137	71.2641	Native vegetation	Cigliano et al. (1989)	October 1972 - Described as *Elasmoderus rabiosus*
36	*Elasmoderus lutescens* (Blanchard, 1851)	Los Molles	Elqui	Coquimbo	29.9783	70.9497	Native vegetation	Data published in this work	October 2011
37	*Elasmoderus lutescens* (Blanchard, 1851)	La Serena	Elqui	Coquimbo	29.8841	71.1928	Dryland and crops	Elgueta et al. (1999)	-
38	*Elasmoderus lutescens* (Blanchard, 1851)	Coquimbo	Elqui	Coquimbo	29.9603	71.3534	Shrub steppe of the foothills	Elgueta et al. (1999), Alfaro et al. (2013)	-
39	*Elasmoderus lutescens* (Blanchard, 1851)	Altovalsol	Elqui	Coquimbo	29.9322	71.1165	Shrub steppe of the foothills	Data published in this work	October 2011
40	*Elasmoderus lutescens* (Blanchard, 1851)	Marquesa	Elqui	Coquimbo	29.9517	70.9708	Dryland and crops	Alfaro et al. (2011)	-
41	*Elasmoderus lutescens* (Blanchard, 1851)	Vicuña	Elqui	Coquimbo	30.0213	70.7089	Dryland and crops	Elgueta et al. (1999)	-
42	*Elasmoderus lutescens* (Blanchard, 1851)	Diaguitas	Elqui	Coquimbo	30.0169	70.6382	Dryland and crops	Alfaro et al. (2011)	-
43	*Elasmoderus lutescens* (Blanchard, 1851)	Andacollito	Elqui	Coquimbo	30.0288	70.6228	Dryland and crops	Data published in this work	October 2008
44	*Elasmoderus lutescens* (Blanchard, 1851)	Puente Negro	Elqui	Coquimbo	30.1011	70.4899	Dryland and crops	Data published in this work	October 2008
45	*Elasmoderus lutescens* (Blanchard, 1851)	Monte Grande	Elqui	Coquimbo	30.0933	70.4968	Dryland and crops	Data published in this work	October 2008
46	*Elasmoderus lutescens* (Blanchard, 1851)	Paihuano	Elqui	Coquimbo	30.0260	70.5269	Dryland and crops	Data published in this work	October 2008
47	*Elasmoderus lutescens* (Blanchard, 1851)	Pisco Elqui	Elqui	Coquimbo	30.1214	70.4994	Dryland and crops	Alfaro et al. (2011)	-
48	*Elasmoderus lutescens* (Blanchard, 1851)	El Pangue	Elqui	Coquimbo	30.1683	70.6650	Dryland and crops	Data published in this work	October 1996
49	*Elasmoderus lutescens* (Blanchard, 1851)	Elqui	Elqui	Coquimbo	29.8428	70.7828	Native vegetation and crops slightly affected	Cigliano et al. (1989)	October 1944
50	*Elasmoderus lutescens* (Blanchard, 1851)	Tongoy	Elqui	Coquimbo	30.2664	71.4782	Dryland and crops	Elgueta et al. (1999)	-
51	*Elasmoderus lutescens* (Blanchard, 1851)	Mineral Las Breas	Elqui	Coquimbo	30.3367	70.9722	Dryland and crops	Cigliano et al. (1989)	december 1956
52	*Elasmoderus lutescens* (Blanchard, 1851)	Corral de Julio	Elqui	Coquimbo	31.1692	71.6192	Dryland and crops	Elgueta et al. (1999)	-
53	*Elasmoderus lutescens* (Blanchard, 1851)	Guánuco	Elqui	Coquimbo	29.4002	71.2306	Native vegetation	Cigliano et al. (1989)	December 1957 - Described as *Elasmoderus rabiosus*
54	*Elasmoderus lutescens* (Blanchard, 1851)	Cerro Las Nipas	Elqui	Coquimbo	30.4530	71.4025	Native vegetation and crops slightly affected	Cepeda-Pizarro et al. (2006)	-

55	*Elasmoderus lutescens* (Blanchard, 1851)	Cuesta de Almendro, El Pangue	Limarí	Coquimbo	31.5545	71.4538	Dryland and crops	Cigliano et al. (1989), Elgueta et al. (1999)	October 1957
56	*Elasmoderus lutescens* (Blanchard, 1851)	Tabali	Limarí	Coquimbo	30.6389	71.4287	Dryland and crops	Data published in this work	October 2015
57	*Elasmoderus lutescens* (Blanchard, 1851)	Camarico	Limarí	Coquimbo	30.7231	71.3339	Dryland and crops	Data published in this work	December 2017
58	*Elasmoderus lutescens* (Blanchard, 1851)	Tuqui, Ovalle	Limarí	Coquimbo	30.5389	71.2169	Dryland and crops	Data published in this work	October 2015
59	*Elasmoderus lutescens* (Blanchard, 1851)	Ovalle	Limarí	Coquimbo	30.6190	71.1870	Dryland and crops	Cigliano et al. (1989), Elgueta et al. (1999)	December 1957
60	*Elasmoderus minutus* Cigliano, Ronderos & Kemp, 1989	Parque Nacional Pan de Azucar	Antofagasta	Atacama	26.1517	70.6428	Coastal desert scrub	Elgueta et al. (1999), Pizarro-Araya and Alfaro (2015)	November 1997
61	*Elasmoderus minutus* Cigliano, Ronderos & Kemp, 1989	Majadas	Copiapó	Atacama	27.2082	70.8934	Coastal desert scrub	Cigliano et al. (1989), Elgueta et al. (1999), Donato (2000), Pizarro-Araya and Alfaro (2015)	December 1965
62	*Elasmoderus minutus* Cigliano, Ronderos & Kemp, 1989	Norte de Castilla	Copiapó	Atacama	27.7945	70.6410	Coastal desert scrub	Cigliano et al. (1989), Pizarro-Araya and Alfaro (2015)	November 1972
63	*Elasmoderus minutus* Cigliano, Ronderos & Kemp, 1989	Castilla	Copiapó	Atacama	27.9046	70.6839	Flowering desert	Cigliano et al. (1989), Elgueta et al. (1999), Pizarro-Araya and Alfaro (2015)	October 1965, December 2014
64	*Elasmoderus minutus* Cigliano, Ronderos & Kemp, 1989	40-60 Km S Copiapó	Copiapó	Atacama	27.8241	70.3375	Flowering desert	Donato (2000), Pizarro-Araya and Alfaro (2015)	November 1972
65	*Elasmoderus minutus* Cigliano, Ronderos & Kemp, 1989	Camino a Pascua Lama	Huasco	Atacama	29.2459	69.5888	High Andean vegetation	Data published in this work	november 2014
66	*Elasmoderus wagenknechti* (Liebermann, 1954)	Loma el Sauce	Limarí	Coquimbo	30.4822	71.3267	Native vegetation and crops slightly affected	Cepeda-Pizarro et al. (2003)	1970, 1996
67	*Elasmoderus wagenknechti* (Liebermann, 1954)	El Higirio	Limarí	Coquimbo	30.4822	71.3267	Xerophytic scrub	Data published in this work	November 1972
68	*Elasmoderus wagenknechti* (Liebermann, 1954)	Cerrillos de Tamaya	Limarí	Coquimbo	30.5799	71.3992	Dryland and crops	Cigliano et al. (1989), Elgueta et al. (1999)	November 1972
69	*Elasmoderus wagenknechti* (Liebermann, 1954)	Fray Jorge	Limarí	Coquimbo	30.6758	71.6656	Dryland and crops	Cigliano et al. (1989), Elgueta et al. (1999)	-
70	*Elasmoderus wagenknechti* (Liebermann, 1954)	Alcones Altos	Limarí	Coquimbo	30.8092	71.5276	Dryland and crops	Data published in this work	September 2015
71	*Elasmoderus wagenknechti* (Liebermann, 1954)	Socos, Alcones	Limarí	Coquimbo	30.7090	71.5001	Dryland and crops	Elgueta et al. (1999)	-
72	*Elasmoderus wagenknechti* (Liebermann, 1954)	Socos	Limarí	Coquimbo	30.7090	71.5001	Xerophytic scrub	Cigliano et al. (1989), Elgueta et al. (1999)	November 1972
73	*Elasmoderus wagenknechti* (Liebermann, 1954)	Camarico Viejo	Limarí	Coquimbo	30.7565	71.3741	Native vegetation, no attack on crops reported	Cepeda-Pizarro et al. (2006)	1970
74	*Elasmoderus wagenknechti* (Liebermann, 1954)	Alcones	Limarí	Coquimbo	30.7853	71.5479	Native vegetation, no attack on crops reported	Cepeda-Pizarro et al. (2006)	1970
75	*Elasmoderus wagenknechti* (Liebermann, 1954)	Agua Amarilla 1	Limarí	Coquimbo	30.8333	70.7000	Dryland and crops	Cepeda-Pizarro et al. (2006)	1996, 1999
76	*Elasmoderus wagenknechti* (Liebermann, 1954)	Agua Amarilla 2	Limarí	Coquimbo	30.8333	70.7000	Native vegetation, no attack on crops reported	Cepeda-Pizarro et al. (2006)	1970, 1974, 1975, 1977
77	*Elasmoderus wagenknechti* (Liebermann, 1954)	Peña Blanca	Limarí	Coquimbo	30.9002	71.5631	Native vegetation	Cepeda-Pizarro et al. (2006)	1974, 1975
78	*Elasmoderus wagenknechti* (Liebermann, 1954)	La Laja	Limarí	Coquimbo	30.9174	71.0279	Native vegetation, no attack on crops reported	Elgueta et al. (1999), Cepeda-Pizarro et al. (2006)	1970
79	*Elasmoderus wagenknechti* (Liebermann, 1954)	Cancha de aterrizaje de Quilitapia	Limarí	Coquimbo	30.9236	71.2611	Native vegetation, no attack on crops reported	Cepeda-Pizarro et al. (2006)	October 1996
80	*Elasmoderus wagenknechti* (Liebermann, 1954)	Entre San Marcos y Cogotí	Limarí	Coquimbo	30.9851	71.0868	Native vegetation	Cigliano et al. (1989)	November 1972
81	*Elasmoderus wagenknechti* (Liebermann, 1954)	Cogotí	Limarí	Coquimbo	31.0161	71.1048	Dryland and crops	Elgueta et al. (1999), Cepeda-Pizarro et al. (2006)	October 1996, 1999
82	*Elasmoderus wagenknechti* (Liebermann, 1954)	Andacollito del Huacho	Limarí	Coquimbo	31.0298	71.1746	Dryland and crops	Cepeda-Pizarro et al. (2006)	October 1996, 1999

83	*Elasmoderus wagenknechti* (Liebermann, 1954)	Cruz Grande del Huacho	Limarí	Coquimbo	31.0298	71.1746	Dryland and crops	Cepeda-Pizarro et al. (2006)	October 1996, 1999
84	*Elasmoderus wagenknechti* (Liebermann, 1954)	Quebrada del Huacho	Limarí	Coquimbo	31.0298	71.1746	Dryland and crops	Cepeda-Pizarro et al. (2006)	October 1996, 1999
85	*Elasmoderus wagenknechti* (Liebermann, 1954)	El Huacho	Limarí	Coquimbo	31.0302	71.1821	Native vegetation, no attack on crops reported	Cepeda-Pizarro et al. (2006)	1970, 1972, 1974, 1980, 1999
86	*Elasmoderus wagenknechti* (Liebermann, 1954)	Sauce Bajo	Limarí	Coquimbo	31.0744	71.1357	Native vegetation	Cepeda-Pizarro et al. (2006)	1975, October 1999
87	*Elasmoderus wagenknechti* (Liebermann, 1954)	Quebrada de Lepe	Limarí	Coquimbo	31.1036	71.1692	Dryland and crops	Cepeda-Pizarro et al. (2006)	1974, 1975
88	*Elasmoderus wagenknechti* (Liebermann, 1954)	Estacion La Mostaza	Limarí	Coquimbo	31.1066	71.0433	Native vegetation, no attack on crops reported	Cepeda-Pizarro et al. (2006)	1970
89	*Elasmoderus wagenknechti* (Liebermann, 1954)	Soruco	Limarí	Coquimbo	31.1070	71.1083	Dryland and crops	Cepeda-Pizarro et al. (2006)	1977, 1980
90	*Elasmoderus wagenknechti* (Liebermann, 1954)	Chipel	Limarí	Coquimbo	31.1097	71.1962	Dryland and crops	Cepeda-Pizarro et al. (2006)	1970, 1974, 1975
91	*Elasmoderus wagenknechti* (Liebermann, 1954)	Quilitapia	Limarí	Coquimbo	31.1162	71.0754	Dryland and crops	Cigliano et al. (1989), Elgueta et al. (1999), Cepeda-Pizarro et al. (2006)	1970
92	*Elasmoderus wagenknechti* (Liebermann, 1954)	Quilitapia, Divisadero, Camino a Carmencita, Sector 1	Limarí	Coquimbo	31.1227	71.1747	Thorny steppe scrub	Data published in this work	November 2022
93	*Elasmoderus wagenknechti* (Liebermann, 1954)	Quilitapia, Divisadero, Camino a Carmencita, Sector 2	Limarí	Coquimbo	31.1275	71.1859	Thorny steppe scrub	Data published in this work	November 2022
94	*Elasmoderus wagenknechti* (Liebermann, 1954)	Quilitapia, Divisadero, Camino a Carmencita, Sector 3	Limarí	Coquimbo	31.1300	71.1888	Thorny steppe scrub	Data published in this work	November 2022
95	*Elasmoderus wagenknechti* (Liebermann, 1954)	Quilitapia, Divisadero, Camino a Carmencita, Sector 4	Limarí	Coquimbo	31.1583	71.1971	Thorny steppe scrub	Data published in this work	November 2022
96	*Elasmoderus wagenknechti* (Liebermann, 1954)	Quilitapia, Divisadero, Camino a Carmencita, Sector 5	Limarí	Coquimbo	31.9193	71.1216	Thorny steppe scrub	Data published in this work	November 2022
97	*Elasmoderus wagenknechti* (Liebermann, 1954)	Quilitapia, Divisadero, Camino a Carmencita, Sector 6	Limarí	Coquimbo	31.1551	71.1979	Thorny steppe scrub	Data published in this work	November 2022
98	*Elasmoderus wagenknechti* (Liebermann, 1954)	Divisadero	Limarí	Coquimbo	31.9180	71.1153	Native vegetation, no attack on crops reported	Cepeda-Pizarro et al. (2006)	1970
99	*Elasmoderus wagenknechti* (Liebermann, 1954)	Combarbalá	Limarí	Coquimbo	31.1764	71.0167	Interior dryland vegetation	Cigliano et al. (1989), Elgueta et al. (1999)	1970
100	*Elasmoderus wagenknechti* (Liebermann, 1954)	La Capilla (Combarbalá)	Limarí	Coquimbo	31.2041	71.0693	Interior dryland vegetation	Data published in this work	November 2005
101	*Elasmoderus wagenknechti* (Liebermann, 1954)	Placa (Combarbalá)	Limarí	Coquimbo	31.1800	71.0111	Interior dryland vegetation	Cepeda-Pizarro et al. (2006)	1980
102	*Elasmoderus wagenknechti* (Liebermann, 1954)	Chuchini (Combarbalá)	Limarí	Coquimbo	31.1007	71.0453	Interior dryland vegetation	Data published in this work	September 2011
103	*Elasmoderus wagenknechti* (Liebermann, 1954)	Sauce Bajo	Limarí	Coquimbo	31.0744	71.1357	Interior dryland vegetation	Cepeda-Pizarro et al. (2006)	1975
104	*Elasmoderus wagenknechti* (Liebermann, 1954)	Comunidad Jiménez y Tapia	Limarí	Coquimbo	31.1794	71.0098	Interior dryland vegetation	Cepeda-Pizarro et al. (2006)	1974
105	*Elasmoderus wagenknechti* (Liebermann, 1954)	Aeródromo de Chingay	Limarí	Coquimbo	31.2236	71.0740	Native vegetation, no attack on crops reported	Cepeda-Pizarro et al. (2006)	1970

106	*Elasmoderus wagenknechti* (Liebermann, 1954)	Pama Arriba	Limarí	Coquimbo	31.2375	71.0301	Interior dryland vegetation	Cepeda-Pizarro et al. (2006)	1980
107	*Elasmoderus wagenknechti* (Liebermann, 1954)	Pama 1	Limarí	Coquimbo	31.2350	71.0427	Native vegetation, no attack on crops reported	Cepeda-Pizarro et al. (2006)	1970
108	*Elasmoderus wagenknechti* (Liebermann, 1954)	Pama 2	Limarí	Coquimbo	31.2351	71.0437	Xerophytic scrub	Cepeda-Pizarro et al. (2006)	1972
109	*Elasmoderus wagenknechti* (Liebermann, 1954)	Chingay	Limarí	Coquimbo	31.2420	71.0998	Xerophytic scrub	Cepeda-Pizarro et al. (2006)	1972, 1974, 1977, 1980
110	*Elasmoderus wagenknechti* (Liebermann, 1954)	Blanquillo	Limarí	Coquimbo	31.2501	71.0641	Interior dryland vegetation	Cepeda-Pizarro et al. (2006)	1980
111	*Elasmoderus wagenknechti* (Liebermann, 1954)	Punitaqui	Limarí	Coquimbo	31.8251	71.2621	Interior dryland vegetation	Data published in this work	October 2015
112	*Elasmoderus wagenknechti* (Liebermann, 1954)	Illapel	Choapa	Coquimbo	31.6196	71.1642	Interior dryland vegetation	Cigliano et al. (1989)	October 1954 - Described as *Elasmoderus rabiosus*

References – Supplemental Table S1

Alfaro, F.M.; Pizarro-Araya, J.; Cepeda-Pizarro, J.; Bodini, A., 2011. Composición y distribución del ensamble de Orthoptera (Insecta) en una cuenca árida del norte-centro de Chile. Rev. Fac. Cienc. Agrar. 43, 97–110.

Alfaro, F.M.; Pizarro-Araya, J.; Letelier, L.; Cepeda-Pizarro, J., 2013. Distribución geográfica de los ortópteros (Insecta: Orthoptera) presentes en las provincias biogeográficas de Atacama y Coquimbo (Chile). Rev. de Geogr. Norte Gd. 56, 235–250. https://doi.org/10.4067/S0718-34022013000300013

Cepeda-Pizarro, J.; Vega, S.; Elgueta, M.; Pizarro-Araya, J., 2006. Algunos antecedentes meteorológicos que explican las irrupciones poblacionales de *Elasmoderus wagenknechti* (Liebermann) (Orthoptera: Tristiridae) en la región del semiárido de Chile. Idesia. 24, 49–64. https://doi.org/10.4067/S0718-34292006000300007

Cepeda-Pizarro, J.; Vega, S.; Vásquez, H.; Elgueta, M., 2003. Morfometría y dimorfismo sexual de *Elasmoderus wagenknechti* (Liebermann) (Orthoptera: Tristiridae) en dos eventos de irrupción poblacional. Rev. Chil. Hist. Nat. 76, 417–435. https://doi.org/10.4067/S0716-078X2003000300007

Cigliano, M.M.; Ronderos, R.A.; Kemp, W.P., 1989. Revision of the genus *Elasmoderus* Saussure (Orthoptera: Tristiridae). Can. Entomol. 121, 225–243. https://doi.org/10.4039/Ent121225-3

Donato, M., 2006. Historical biogeography of the family Tristiridae (Orthoptera: Acridomorpha) applying dispersal-vicariance analysis. J. Arid Environ. 66, 421–434.

Elgueta, M.; Camousseight, A.; Carbonell, C. 1999. Catálogo de los Orthoptera (Insecta) de Chile. Publ. Ocas. Mus. Nac. Hist. Nat. 54, 1–60. https://doi.org/10.1016/j.jaridenv.2006.01.006

Pizarro-Araya, J.; Alfaro, F.M. 2015. *Elasmoderus minutus* Cigliano Ronderos & Kemp, 1989 (Orthoptera: Tristiridae). In: 11º Proceso de Clasificación de Especies (DS 00/2015 MMA). Ministerio del Medio Ambiente, Chile. Vulnerable VU B1ab(iii)+2ab(iii). Available online: https://clasificacionespecies.mma.gob.cl/wp-content/uploads/2019/10/Elasmoderus_minutus_11RCE_04_PAC.pdf (accessed on 01 April 2024).
